# The Combination of Paraformaldehyde and Glutaraldehyde Is a Potential Fixative for Mitochondria

**DOI:** 10.3390/biom11050711

**Published:** 2021-05-10

**Authors:** Yuan Qin, Wenting Jiang, Anqi Li, Meng Gao, Hanyu Liu, Yufei Gao, Xiangang Tian, Guohua Gong

**Affiliations:** 1 Institute for Regenerative Medicine, Shanghai East Hospital, School of Life Sciences and Technology, Tongji University, Shanghai 200092, China; yuan_q19@163.com (Y.Q.); hggjoy@163.com (W.J.); aq.li@outlook.com (A.L.); meng_gao1993@163.com (M.G.); hanyuliu1997@163.com (H.L.); yufeigao2021@163.com (Y.G.); 2 Department of Pharmacy, Shanghai East Hospital, Tongji University, Shanghai 200120, China; 3 Department of Cardiovascular Surgery, Daping Hospital, Army Medical Center of PLA, Chongqing 400037, China; xgangtian@outlook.com

**Keywords:** mitochondria, fixative, mitochondrial morphology, paraformaldehyde, glutaraldehyde

## Abstract

Mitochondria are highly dynamic organelles, constantly undergoing shape changes, which are controlled by mitochondrial movement, fusion, and fission. Mitochondria play a pivotal role in various cellular processes under physiological and pathological conditions, including metabolism, superoxide generation, calcium homeostasis, and apoptosis. Abnormal mitochondrial morphology and mitochondrial protein expression are always closely related to the health status of cells. Analysis of mitochondrial morphology and mitochondrial protein expression in situ is widely used to reflect the abnormality of cell function in the chemical fixed sample. Paraformaldehyde (PFA), the most commonly used fixative in cellular immunostaining, still has disadvantages, including loss of antigenicity and disruption of morphology during fixation. We tested the effect of ethanol (ETHO), PFA, and glutaraldehyde (GA) fixation on cellular mitochondria. The results showed that 3% PFA and 1.5% GA (PFA-GA) combination reserved mitochondrial morphology better than them alone in situ in cells. Mitochondrial network and protein antigenicity were well maintained, indicated by preserved MitoTracker and mitochondrial immunostaining after PFA-GA fixation. Our results suggest that the PFA-GA combination is a valuable fixative for the study of mitochondria in situ.

## 1. Introduction

Mitochondria are major highly dynamic organelles regulated by fission and fusion. Mitochondrial size, shape, and location are variable in different cell types [1,2,3,4]. Mitochondria autonomously respond to energy demands and environmental changes in cells by reshaping morphology [4,5,6]. The morphology of mitochondria is directly related to the functions of cells and tissues [7,8,9,10]. In most cases, mitochondria in live cells can be directly visualized by microscope technology [11,12]. However, in some cases, cell samples cannot be imaged immediately after collection, thus they have to be frozen in a specific state using chemical fixation [13,14]. Then, the preserved mitochondria can be visualized by immunocytochemistry or confocal microscopy.

Fixation can inhibit cell autolysis, retain cell components, maintain cell morphology and structural integrity, and make the microscopic appearance of cells more apparent [15]. The fixing method can be divided into two types: cross-linking and denaturation [16]. The cross-linking fixatives contain various aldehydes, such as paraformaldehyde (PFA) and glutaraldehyde (GA). In tissues, PFA can quickly cross-link proteins over a short range, while GA can slowly cross-link proteins over a long distance [17,18,19,20]. However, in cultured cells, the speed of GA fixation is much faster than PFA [21]. Denaturing fixatives, also known as precipitating fixatives, are commonly used, such as ethanol and methanol. Ethanol can reduce the solubility of proteins in cells, destroy the hydrophobic interactions between proteins, and coagulate the proteins [22]. The disadvantages of the different fixatives are also apparent. There exists severe cell shrinkage in ethanol fixation. PFA, as the most commonly used fixative for immunostaining of cells, has been associated with various problems, including loss of antigenicity and disruption of morphology during fixation [23,24].

Recently, accumulating studies have been reported to explore more efficient fixation methods in different cells or tissues [16,22,25,26]. However, previous research only focuses on the effects of different fixation methods at a cellular level [27,28,29]. They ignored the influences of fixation methods on the visualization of mitochondria. Therefore, more effort is needed to study the methods of fixing mitochondria.

In this study, we investigated the effects of different fixatives, including ethanol (ETHO), PFA, and GA, on mitochondria of mouse embryonic fibroblasts (MEFs). We monitored the morphology and protein antigenicity of mitochondria before and after fixation. Our results show that the combination of 3% PFA/1.5% GA (PFA-GA) could retain the morphology and protein antigenicity of mitochondria in cells.

## 2. Materials and Methods

### 2.1. Cell Culture

Mouse embryo fibroblasts (MEFs) were cultured in Dulbecco’s Modified Eagle Medium (DMEM, Gibco, Grand Island, NY, USA) supplemented with 10% fetal bovine serum, 1% penicillin/streptomycin, and 1% non-essential amino acids and were maintained at 37 °C and 5% CO_2_. All of the cells in this study were maintained between passages 3 and 5.

### 2.2. Chemical Fixation

After being washed with PBS, cells were stained with 200 nM MitoTracker Red (ThermoFisher Scientific, Carlsbad, CA, USA) for 30 min at 37 °C for labeling mitochondria. Washed twice with PBS and read the fluorescence intensity using a microplate reader. Then, the different chemical fixatives (Table S1) were quickly added to each well. The change of fluorescence intensity was continuously recorded at 1-minute intervals.

### 2.3. Confocal Imaging

Cell imaging used a Zeiss LSM 800 confocal microscope equipped with a 60× 1.3 NA oil immersion objective and followed a procedure developed previously [30]. Approximately 1 × 10^5^ cells were plated on a 25 mm diameter coverslip in 6-well plates and cultured at 37 °C in a 5% CO_2_ incubator for 24 h. Cells were stained with 200 nM MitoTracker Red or 100 nM tetramethylrhodamine ethyl ester (TMRE) (ThermoFisher Scientific, Carlsbad, CA, USA) for 30 min at 37 °C for labeling mitochondria. Then, cells were washed twice with KHB, and fixed with 4% formaldehyde, 95% ethanol, 2.5% glutaraldehyde, or the mixture of 3% formaldehyde and 2.5% glutaraldehyde for 10 min at room temperature, respectively. For membrane permeabilization, 0.1% Triton X-100 (Sigma, St. Louis, MO, USA) was applied for 5 min following fixation. All images were acquired at 543, or/and 488 nm laser excitation.

### 2.4. RNA Isolation

Approximately 5 × 10^5^ MEFs were plated in 100 mm dishes and cultured at 37 °C in a 5% CO_2_ incubator for 24 h. The fixed cells were immediately washed in PBS with 1:1000 RNase Inhibitor (Invitrogen, Carlsbad, CA, USA). After collecting by cell scraper, cells were pelleted by centrifugation at 5000× *g* for 5 min at 4 °C. The supernatant was discarded. Total RNA was isolated from the pellet fixed by 4% formaldehyde, 2.5% glutaraldehyde, and 3% formaldehyde/1.5% glutaraldehyde using the RecoverAll Total Nucleic Acid Isolation kit (Invitrogen), starting at the protease digestion stage of the manufacturer-recommended protocol. Total RNA was isolated from the pellet fixed by 95% ethanol and unfixed using the GeneJET RNA Purification Kit (ThermoFisher Scientific) following the manufacturer’s protocol.

### 2.5. Quantitative RT-PCR

HiScript III RT SuperMix for qPCR (+gDNA wiper) (Vazyme, Nangjing, China) was used to synthesize cDNA from 0.3 μg of total RNA.

RNA expression analysis was performed using SYBR Green PCR master mix (4309155, Applied Biosystems, Foster City, CA, USA) with designed primers (Table S2).

### 2.6. Protein Isolation

Approximately 5 × 10^5^ MEFs were plated in 100 mm dishes and cultured at 37 °C in a 5% CO_2_ incubator for 24 h. MEFs fixed by 4% formaldehyde, 2.5% glutaraldehyde, 95% ethanol, and 3% formaldehyde/1.5% glutaraldehyde were washed with PBS. After collecting by cell scraper, cells were pelleted by centrifugation at 5000× *g* for 5 min at 4 °C. The supernatant was discarded. Fixed and non-fixed cells were dissolved in lysis buffer 300 mM Tris (Macklin, Shanghai, China) at pH 8, with 2% SDS (VETEC, St. Louis, MO, USA) and a final concentration of 1% Protease Inhibitor (Millipore, Billerica, MA, USA) with an electric homogenizer. The cell pellet fixed by 4% formaldehyde and 2.5% glutaraldehyde were treated 30 min at 100 °C followed by 2 h at 60 °C. The cell pellet fixed by 95% ethanol and unfixed were treated 30 min on ice. Moreover, all samples were centrifuged at 4 °C for 20 min at 14,000 rpm, and supernatants were transferred to fresh microcentrifuge tubes.

### 2.7. Western Blot

The protein concentration was measured using the BCA assay (ThermoFisher Scientific, Carlsbad, CA, USA). 20 µg protein per sample was used for SDA-PAGE. The gels were stained in Coomassie brilliant blue for 1 h and de-stained in a 50% ethanol, 40% ddH_2_O, and 10% acetic acid mixture for 3–4 h. The stained gels were scanned and analyzed using an automatic digital gel image analysis system (Tanon, Shanghai, China) following the manufacturer’s guidelines.

To detect mitochondrial Mfn2, 20 µg protein per sample was used for SDA-PAGE. Then, the separated proteins were electro-transferred onto Immobilon polyvinylidene fluoride (PVDF) membranes (Millipore, Billerica, MA, USA). The membrane was blocked at room temperature for 1 h with 5% milk powder in PBST (PBS, 0.05% Tween 20) and incubated with primary antibodies specific to GAPDH (BBI, Shanghai, China, D110016) and mfn2 (Abcam, Cambridge, UK, ab6789) overnight at 4 °C. Then, membranes were washed 3 times in PBST for 10 min each and incubated with goat anti-mouse (Abcam; ab56889) or anti-rabbit secondary antibody (Pierce, Rockford, IL, USA) at room temperature for 1 h on a shaker. Membranes were washed three times in PBST for 10 min each again. Protein band intensities were developed using High-sig ECL Western Blotting Luminol/Enhancer solution (Tanon, Shanghai, China) and measured using a chemiluminescence imaging system (CLiNX, Shanghai, China) following the manufacturer’s guidelines.

### 2.8. Immunofluorescent Labeling

Approximately 1 × 10^5^ MEFs were plated in chambers and cultured at 37 °C in a 5% CO_2_ incubator for 48 h. Then, cells were washed twice with PBS. Fixed cells with 4% formaldehyde, 95% ethanol, 2.5% glutaraldehyde, or the mixture of 3% formaldehyde and 1.5% glutaraldehyde for 20 min at room temperature, respectively. The coverslips were heated in antigen retrieval buffer (100 mM Tris, 5% (*w*/*v*) urea, pH 9.5) at 95 °C for 10 min. Following fixation, cells were washed 3 times in PBS and permeabilized with 0.1% Triton X-100 in PBS for 10 min. Cells were washed twice for 5 min and blocked with 8% goat serum in PBS for 1 h. Primary antibodies VADC1 (5 μg/mL) and COX IV (10 μg/mL) (Abcam) were incubated overnight at 4 °C in 2% goat serum in PBS after washed once in PBS for 5 min. Then cells were washed in 1% goat serum, 0.1% Tween-20 in PBS 4 times for 10 min. Second antibodies (2 μg/mL) (Invitrogen) were incubated overnight at 4 °C in 2% goat serum in PBS for 1 h after washed in 1% goat serum, 0.1% Tween-20 (Sigma) in PBS 4 times for 10 min. Lastly, added a small amount of ProLong Gold antifade reagent with DAPI (Invitrogen) over the area where each chamber used to be. Cell imaging used a Zeiss LSM 800 confocal microscope (Zeiss, Oberkochen, Germany) equipped with a 60× 1.3 NA oil immersion objective.

### 2.9. Mitochondrial Image Analysis

Mitochondrial aspect ratio (long axis/short axis) was calculated using ImageJ software [31]. Mitochondrial networks and branches were quantified by ImageJ software with a MINA plugin [32]. MiNA enhances the image quality, providing more accurate results. Choices for image pre-processing, including ‘unsharp mask’, CLAHE, and median filtering, are presented to the user through the MiNA interface.

### 2.10. Statistical Analysis

Data represent the mean ± s.e.m of experiments. Statistical comparisons used unpaired Student’s *t*-test or one-way ANOVA followed by Bonferroni’s corrections, as appropriate. *p* < 0.05 was considered statistically significant.

## 3. Results

### 3.1. The Effect of Fixatives on Cells and Mitochondrial Indicators

The commonly used fixatives, including ETHO, PFA, and GA, were applied to cultured MEFs fixation. We first detected the effect of fixative on cell morphology before and after fixation. 95% ethanol (ETHO) caused cells shrinkage after 10 min fixation. Thus, we measured the cell area and found that ETHO significantly decreased cell size (Figure S1). PFA and GA could well maintain the morphology and area of fixed cells.

**Figure 1:**
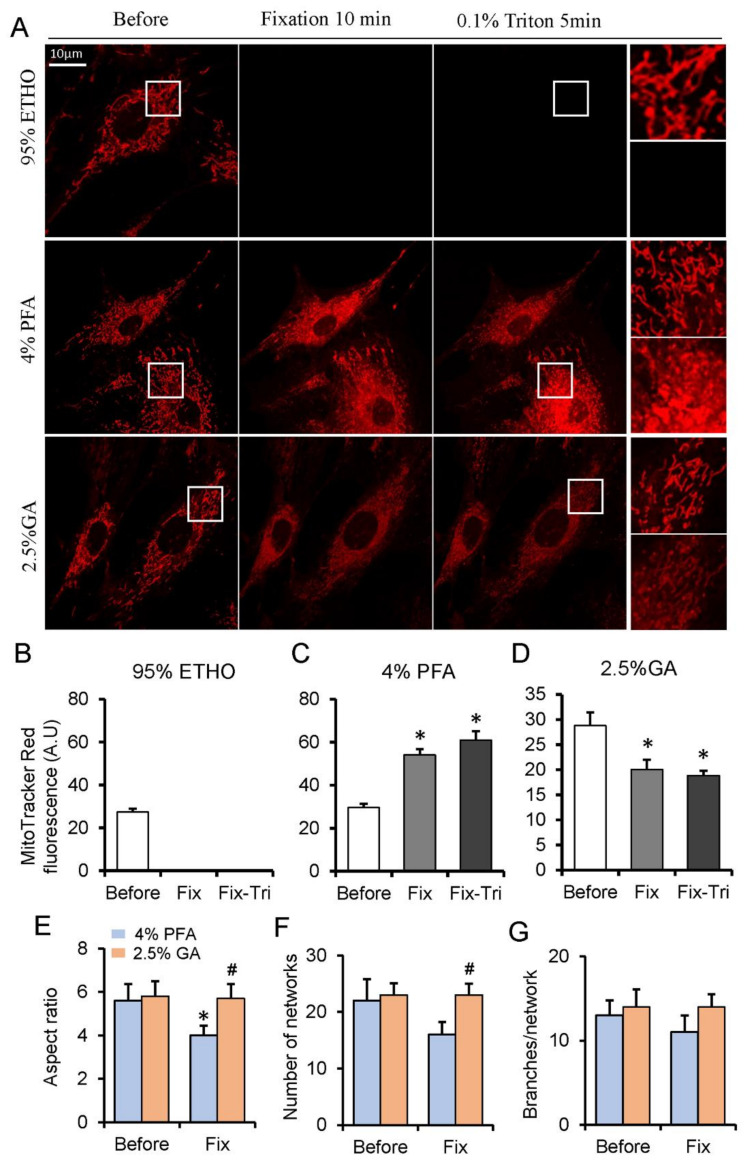
MitoTracker Red fluorescence assay on fixed and permeabilized cells. (**A**) Representative images of the MitoTracker Red fluorescence after fixed by paraformaldehyde (PFA), glutaraldehyde (GA), or ethanol (ETHO), and permeabilized by Triton. (**B**–**D**) Quantity result of MitoTraker Red fluorescence before and after fixed by ETHO, PFA or GA, and permeabilization. (**E**–**G**) Quantitative result of mitochondrial morphology and network. Fix, fixation; Fix-Tri, fixation followed with Triton. Mean ± SEM, n = 9–12 cells. * *p* < 0.05, compared with Before; ^#^ *p* < 0.05, compared with 4% PFA Fix. The data analyzed by one-way ANOVA.

The mitochondria within live cells were generally visualized through the mitochondrial dyes that can accumulate into the mitochondrial matrix. Among them, the MitoTracker Red probes contain a mildly thiol-reactive chloromethyl moiety, which can be retained in mitochondria after fixation [33]. To investigate the effect of fixative on mitochondria, we loaded MEFs with Mitotracker Red. Generally, 20–30 min was applied for ETHO, PFA, or GA fixation [17,21], but we found that the fluorescence of MitoTracker had no apparent difference at 20 min PFA fixation (Figure S2). Given the shorter fixation time of GA and ETHO in cells, we presented 10 min fixation for all fixatives. ETHO led to the fluorescence signal of mitochondria disappearing quickly (Figure 1A,B). PFA increased the fluorescence signal of some mitochondria, and it also triggered MitoTracker Red releasing into cytosol indicated by the fluorescence around the nuclear (Figure 1A,C). No apparent detectable autofluorescence of cells at 543 nm excitation (Figure S3) under the same conditions and at the same scale further confirmed that MitoTracker Red was released into the cytosol (Figure S3B). Triton treatment further increased the fluorescence of mitochondria aggregated around the nuclear (Figure 1A,C). GA led to the fluorescence signal of mitochondria slightly decrease caused by MitoTracker releasing into the cytosol but better maintained the mitochondrial morphology and network (Figure 1E–G). Triton treatment does not further reduce mitochondrial fluorescence (Figure 1A,D). These results indicated that PFA could maintain the signal of MitoTracker Red, and GA could reserve mitochondrial network.

TMRE (Tetramethylrhodamine ethyl ester perchlorate) as a detector of mitochondrial membrane potential is very sensitive to mitochondrial damage. Its’ fluorescence will rapidly disappear after mitochondrial depolarization. To investigate the effect of fixative on mitochondrial damage (membrane potential loss), we loaded MEFs with a mitochondrial membrane potential indicator TMRE. ETHO led to a quick disappearance of fluorescence signals within the mitochondria (Figure S4A,B). PFA slightly decreased the fluorescence signal of mitochondria by triggering TMRE releasing into the cytosol in 1 min but collapsed the TMRE signal after 10 min (Figure S4A,C). GA significantly decreased the fluorescence signal of mitochondria through releasing TMRE into cytosol and solution quickly (Figure S4A,D). These results indicated that no fixative could maintain the signal of mitochondrial membrane potential indicator.

### 3.2. The Effect of Fixative on Cross-Linking Proteins

PFA and GA are cross-linking fixatives that contain a variety of aldehydes. Their high protein cross-link activity results in a protein extraction that is very difficult after fixation. To extract the total protein of fixed cells, we first decross-linked the fixed cells at 100 °C for 30 min, followed by 60 °C 2 h treatment. The still cross-linked proteins will not contribute to the amount of signal in the measurement. ETHO and PFA treatment led to slight protein loss without significant change.

**Figure 2:**
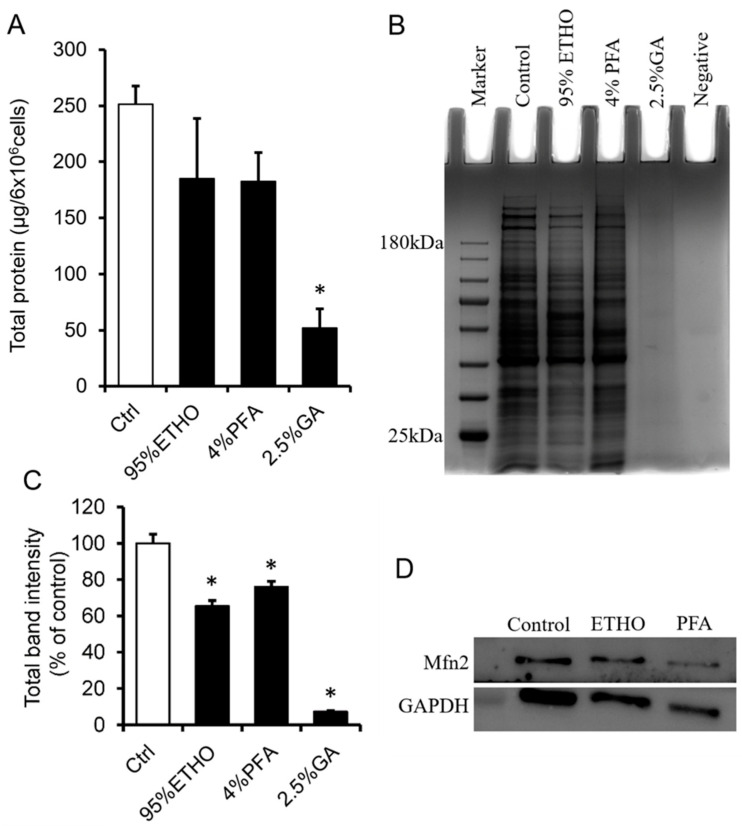
Protein extraction and analysis after fixation. (**A**) The total proteins extracted from fixed cells by ETHO, PFA, or GA. Mean ± SEM, n = 3. * *p* < 0.05. (**B**) Representative images of the SDA-PAGE gel. (**C**) Quantity data of the bands of total protein in PAGE gel. Mean ± SEM, n = 3. * *p* < 0.05. The data analyzed by one-way ANOVA. (**D**) Western blot of Mfn2 and GAPDH after fixed by ETHO and PFA.

GA showed the most robust cross-linking ability, which significantly blocked the extraction of total proteins (Figure 2A). SDS-PAGE showed that the bands of total protein of ETHO and PFA fixed cells are clearly accompanied by slight protein loss (Figure 2B). Western blot could also detect the mitochondrial membrane protein Mfn2 (Figure 2D), but the quantity data of band intensity showed that ETHO, PFA, and GA decreased protein level (Figure 2C). There was no obvious protein band from GA-treated cells (Figure 2B,C). These results indicated that GA had the best fixation ability (protein cross-link activity) for fast freezing protein in situ. It might be a valuable fixative to fix mitochondria for immunostaining assay.

### 3.3. The Screening of More Suitable Fixative for Mitochondria

**Figure 3:**
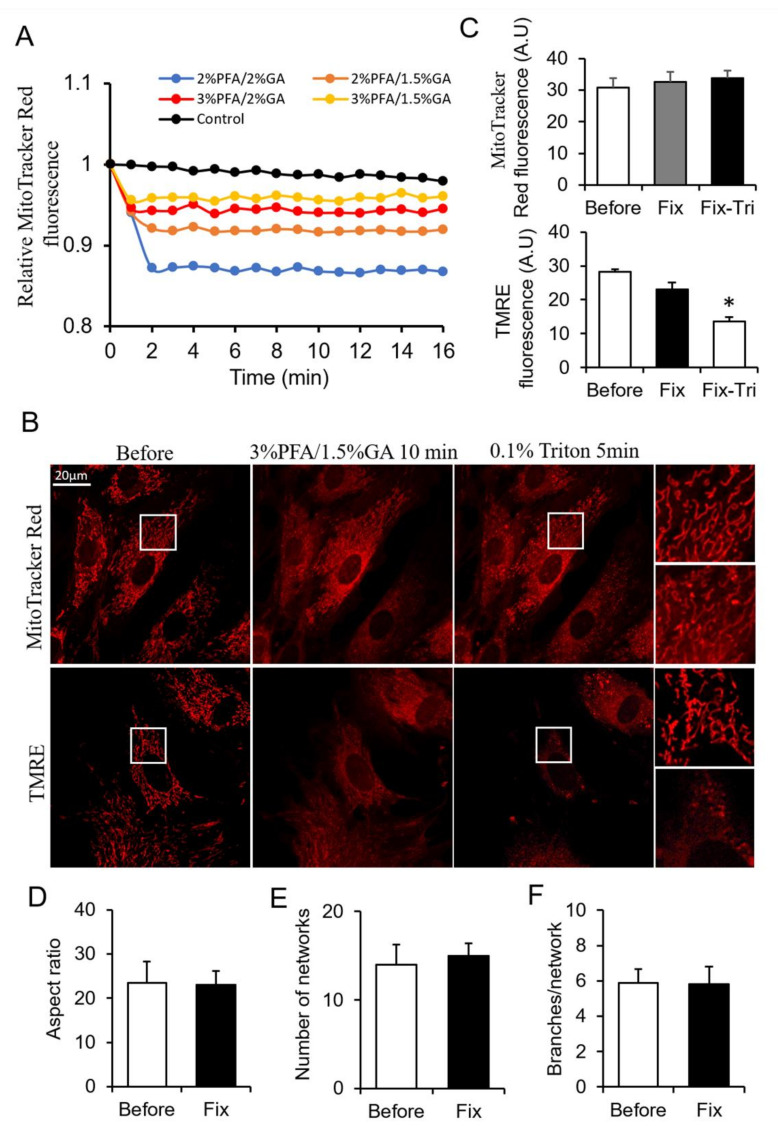
Screening the ratio of PFA and GA. (**A**) Representative traces of the MitoTracker Red fluorescence during fixation. (**B**) Representative images of the MitoTracker Red and TMRE fluorescence after fixed by PFA-GA, and permeabilized by Triton. (**C**) Quantitive data of MitoTracker Red and TMRE fluorescence. (**D**–**F**) Quantitative result of mitochondrial morphology and network using MitoTracker images. Fix, fixation; Fix-Tri, fixation followed with Triton. Mean ± SEM, n = 9–12 cells. * *p* < 0.05. The data analyzed by one-way ANOVA.

From the above results, PFA and GA showed their different merits on mitochondrial fixation. The combination use of PFA and GA is a potential method for better mitochondrial fixation. To obtain a more suitable fixative for mitochondria, we screened tens of PFA and GA combinations by monitoring the change of MitoTracker Red fluorescence during fixation. Here, we showed typical combinations (Figure 3A). The combinations of 3% PFA/1.5% GA showed better fluorescence maintenance than other combinations (Figure 3A). We chose 3% PFA/1.5% GA for further investigation. 3% PFA/1.5% GA maintained most of MitoTracker signal in mitochondria (Figure 3B,C) beside the redistribution of MitoTracker (The perinuclear mitochondria with higher MitoTracker) and mitochondrial morphology and network after fixation (Figure 3D–F). MitoTracker aggregates triggered by triton remarkedly decreased compared with 4% PFA fixation (Figure 1A and Figure 3B). Most of TMRE was kept in the mitochondria, but released TMRE increased the cytosolic background. The result did not indicate that mitochondria were polarized and TMRE was active because further FCCP + Antimycin A treatment did not trigger massive loss of TMRE (Figure S6). Triton treatment further induced the release of TMRE (Figure 3B,C). These results suggested that 3% PFA/1.5% GA was a potential combined fixative for mitochondrial fixation.

The level of extracted total protein was only one-eighth of control after 3% PFA/1.5% GA fixation due to the robust cross-linking ability of GA (Figure S5A). There was no apparent protein band from 3% PFA/1.5% GA treated cells (Figure S5B,C).

### 3.4. mRNA Level After Fixation

**Figure 4:**
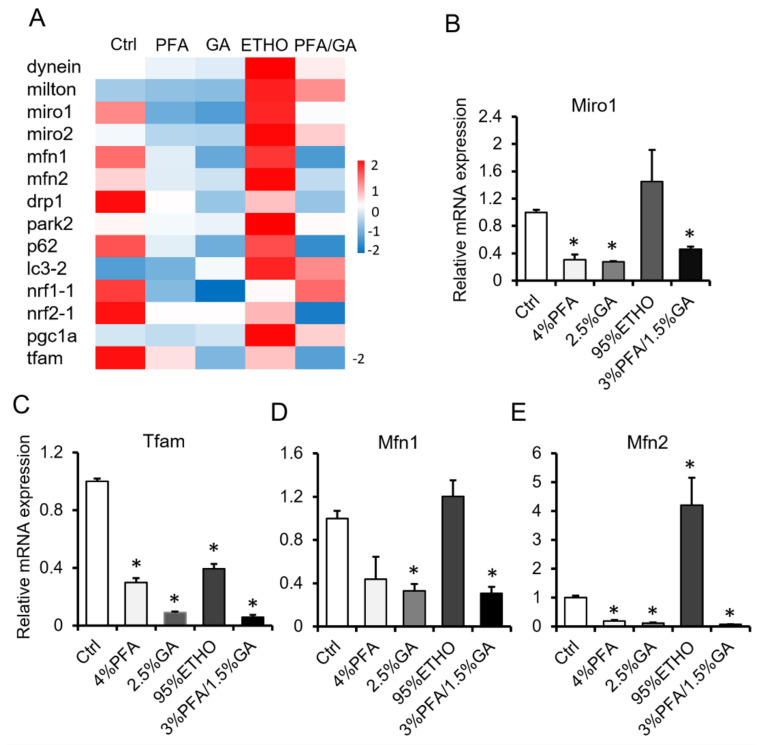
mRNA assay after fixation. (**A**) Hotmap of RNA level after fixed by PFA, GA, ETHO, or PFA-GA. (**B**–**E**) The relative RNA level of Miro1, Tfam, Mfn1, and Mfn2 after fixed by ETHO, PFA, GA, or PFA-GA. Mean ± SEM, n = 3. * *p* < 0.05. The data analyzed by one-way ANOVA.

To confirm the effect of 3% PFA/1.5% GA, we next compared the effect of fixatives on mRNA level. We isolated the total RNA of fixed cells and quantified the RNA. ETHO increased mRNA expression. PFA or GA alone decreased the mRNA level. However, 3% PFA/1.5% GA treatment showed better RNA level reservation, and it even decreased RNA levels (Figure 4). It has been reported that ETHO could up-regulate mitochondrial biogenesis in hepatocytes [34]. Interestingly, ETHO decreased mitochondrial Tfam level but increased Mfn2 level (Figure 4C–E). These results indicated that fixation would affect RNA extraction and measurement.

### 3.5. Immunostaining After Fixation

**Figure 5:**
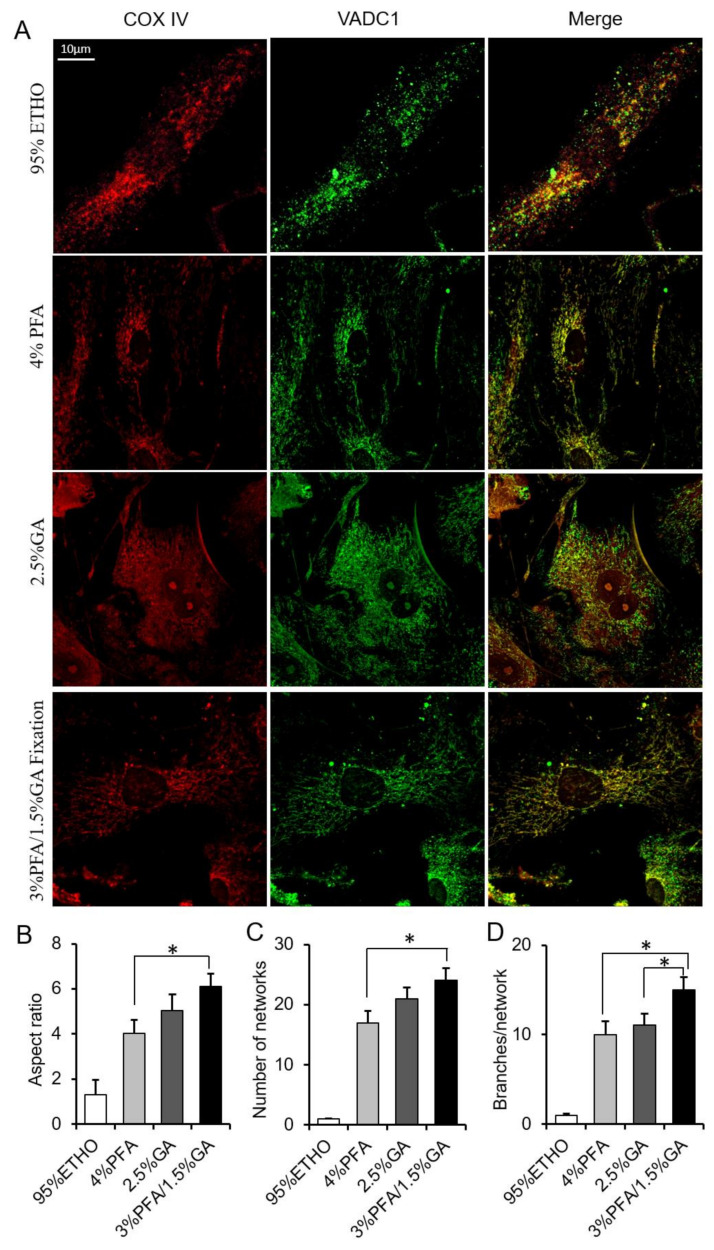
Immunostaining assay after fixation. (**A**) Representative images of the COX IV and VDAC1 immunostaining after fixed by PFA, GA, ETHO, or PFA-GA. (**B**–**D**) Quantitative result of mitochondrial morphology and network. Mean ± SEM, n = 9–12 cells. * *p* < 0.05. The data analyzed by one-way ANOVA.

Next, we further investigated the effect of fixative on mitochondrial morphology and protein antigenicity. Cells were immunostained with VDAC1 and COX IV. The results showed that ETHO damaged the mitochondrial network. 4% PFA maintained most of the mitochondrial network but led to some mitochondrial aggregated around the nuclear, similar to MitoTracker Red staining (Figure 5). 2.5% GA showed a higher background of COX IV and VDAC1. 3% PFA/1.5% GA fixation not only maintained the mitochondrial morphology and network (Figure 5B–D) but also decreased the background of immunostaining (Figure 5A). It is well known that the basis of immunofluorescence is that antibodies specifically recognize antigens. These results indicated that the combination of 3% PFA/1.5% GA (PFA-GA) could retain the morphology and protein antigenicity of mitochondria in cells. Thus, 3% PFA/1.5% GA was the potential fixative for mitochondrial fixation.

## 4. Discussion

Cell fixation is a powerful technique to study the structure and proteins in situ. Mitochondria are constantly undergoing continuous movement in live cells. The study of mitochondrial in situ in the fixed state needs well-maintained mitochondrial morphology and protein antigenicity. The purpose of our study is to compare the effects of various fixations on mitochondria fixation and further find a new method of mitochondrial fixation.

Fixation can inhibit cell autolysis, retain cell components, maintain cell morphology and structural integrity [15]. Generally, the fixing methods can be divided into two typical types: cross-linking and denaturation [16]. The cross-linking fixatives contain a variety of aldehydes, such as formaldehyde, PFA and GA. Different fixatives have their own unique advantages and disadvantages on fixation. PFA cross-links with nearby proteins through its aldehyde groups to form methylene bridge adducts, which can retain the natural distribution of proteins in the cells. However, PFA as a commonly used fixative still has some problems such as the inability to completely fix the proteins during the fixation process, and the induction of the redistribution of the proteins [35], the dissolution of lipid in the cell membranes, the slight damage of the cell membrane’s integrity [19,36,37] and the shield of protein epitopes. While GA, as a linear 5-carbon dialdehyde, can quickly react with the amine group of the protein at around neutral pH [38], it cross-links the protein and maintains a stable structure within a variable distance [18,19,39,40]. In tissues, PFA can quickly cross-link proteins in a short distance, while GA can slowly cross-link proteins over a long distance [17,18,19]. However, in cultured cells, GA fixation is much faster than PFA. 4 min is enough for GA to immobilize the cytoplasmic proteins completely [21,41]. The inadequate fixation will lead to residual mobility, which induced the artificial clustering of receptors [41]. Mitochondria are high dynamic organelle [4,11]. Thus, the quick and robust cross-link ability of GA is beneficial to preserving their antigenicity and morphology. Our data showed that PFA could maintain fluorescence of MitoTracker Red in fixed mitochondria but damage mitochondrial morphology and network; GA could maintain mitochondrial morphology and network but decrease fluorescence of MitoTracker Red. Combining PFA and GA with an appropriate ratio can take advantage of their merits on fixation. It has been found optimal to fix cytoplasmic proteins with at least 1% GA in combination with formaldehyde [21]. Our results also suggested that 1.5% GA in combination with PFA is better for mitochondrial fixation.

Denaturing fixatives, also known as precipitating fixatives, such as methanol, ETHO, and acetone. ETHO can reduce the solubility of proteins in cells, destroy the hydrophobic interactions between proteins and coagulate the proteins. Thence ETHO can modify the tertiary structure of proteins and inactivate enzymes in cells [16]. However, fixation with ETHO can cause severe cell shrinkage due to dehydration, resulting in distortion of the nucleus and cytoplasm details [42,43]. Our data further proved that ETHO fixation damaged mitochondria, including morphology and network. Thus ETHO could not be applied to mitochondrial fixation.

The fixed mitochondria can be used for visualization by immunocytochemistry and confocal microscopy. In over to visualize mitochondrial morphology, mitochondrial dyes can be applied. The MitoTracker Red probes contain a mildly thiol-reactive chloromethyl moiety. The chloromethyl group may retain the dye localization in the mitochondria after fixation [44]. While TMRE is rapidly absorbed by mitochondria and released by fixation, it is mainly used to label living cells. It has been reported that FA-GA fixations are more capable of preserving the overall cellular structures than ETHO and PFA [21,41,43]. Our results also confirmed that MitoTracker Red could be retained into mitochondria after PFA or GA fixation. TMRE was rapidly released from mitochondria after fixation. We found that PFA-GA fixation could retrieve some TMRE signal, such as PFA alone. Further investigation showed FCCP/Antimycin A fail to collapse the TMTE signal, which indicates PFA-GA fixation might not maintain mitochondrial polarization. Immunofluorescence is a technique to detect the distribution of specific proteins in cells and tissues [44]. The fundamental of immunofluorescence is that antibodies specifically recognize antigens. In addition, the cell membrane permeability is also essential for the specific proteins expressed in the cells. Triton X-100, as one of the most commonly used non-ionic detergents, can effectively dissolve cell membranes [45], destroy lipids in membrane proteins, and enhance the penetration of antibodies [46,47]. However, permeabilization with Triton X-100 after formaldehyde treatment induces a decreased cytoplasmic density and apparent loss of organelles because inadequate fixation could not limit the mobilization of cytoplasmic proteins [48]. Thus, employ a rapid, robust fixative, GA, is essential for mitochondrial fixation.

## 5. Conclusions

In summary, our results showed that ETHO could not maintain the mitochondrial network. Thus it is not suitable for mitochondrial fixation. The cross-linking fixative PFA and GA showed different merits when individuals were applied. PFA can maintain the MitoTracker Red signal, GA can better reserve mitochondrial morphology. We found that the appropriate combination of PFA and GA can maintain the MitoTracker Red signal and mitochondrial network. At the same time, the application of GA also avoids the loss of antigenicity caused by PFA. Our study reveals that 3% PFA/1.5% GA is a potential combined fixative for mitochondrial investigation in situ after fixation.

## Data Availability

Not applicable.

## Supplementary Materials

The following are available online at https://www.mdpi.com/article/10.3390/biom11050711/s1, Figure S1: Cell morphology after fixation, Figure S2: Alternation of MitoTracker Red fluorescence after PFA fixed cells, Figure S3: Alternation of autofluorescence in cells after PFA fixed cells, Figure S4: TMRE fluorescence assay on fixed and permeabilized cells, Figure S5: Protein extraction and assay after PFA-GA fixation, Figure S6: Alternation of TMRE fluorescence in cells, Table S1: The combinations of fixatives, Table S2: The sequence of Q-PCR primers.

## References

[1] Soubannier V., McBride H.M. (2009). Positioning mitochondrial plasticity within cellular signaling cascades. Biochim. Biophys. Acta (BBA) Mol. Cell Res..

[2] Qin Y., Li A., Liu B., Jiang W., Gao M., Tian X., Gong G. (2020). Mitochondrial fusion mediated by fusion promotion and fission inhibition directs adult mouse heart function toward a different direction. FASEB J..

[3] El-Hattab A.W., Suleiman J., Almannai M., Scaglia F. (2018). Mitochondrial dynamics: Biological roles, molecular machinery, and related diseases. Mol. Genet. Metab..

[4] Li A., Gao M., Jiang W., Qin Y., Gong G. (2020). Mitochondrial Dynamics in Adult Cardiomyocytes and Heart Diseases. Front. Cell Dev. Biol..

[5] Tepp K., Shevchuk I., Chekulayev V., Timohhina N., Kuznetsov A.V., Guzun R., Saks V., Kaambre T. (2011). High efficiency of energy flux controls within mitochondrial interactosome in cardiac intracellular energetic units. Biochim. Biophys. Acta (BBA) Bioenerg..

[6] Westermann B. (2010). Mitochondrial fusion and fission in cell life and death. Nat. Rev. Mol. Cell Biol..

[7] Dorn Ii G.W. (2015). Mitochondrial dynamism and heart disease: Changing shape and shaping change. EMBO Mol. Med..

[8] Dorn G.W. (2013). Mitochondrial dynamics in heart disease. Biochim. Biophys. Acta (BBA) Mol. Cell Res..

[9] Papanicolaou K.N., Khairallah R.J., Ngoh G.A., Chikando A., Luptak I., Shea K.M., Riley D.D., Lugus J.J., Colucci W.S., Lederer W.J. (2011). Mitofusin-2 Maintains Mitochondrial Structure and Contributes to Stress-Induced Permeability Transition in Cardiac Myocytes. Mol. Cell. Biol..

[10] Rambold A.S., Pearce E.L. (2018). Mitochondrial Dynamics at the Interface of Immune Cell Metabolism and Function. Trends Immunol..

[11] Gong G., Song M., Csordas G., Kelly D.P., Matkovich S.J., Dorn G.W. (2015). Parkin-mediated mitophagy directs perinatal cardiac metabolic maturation in mice. Science.

[12] Qin Y., Gao M., Li A., Sun J., Liu B., Gong G. (2018). Mitoflash lights single mitochondrial dynamics events in mature cardiomyocytes. Biochem. Biophys. Res. Commun..

[13] Chen Y., Dorn G.W. (2013). PINK1-phosphorylated mitofusin 2 is a Parkin receptor for culling damaged mitochondria. Science.

[14] Song J., Lee C., Lin C.-H., Chen L.B. (1991). Electron microscopic studies of the ER in whole-mount cultured cells fixed with potassium permanganate. J. Struct. Biol..

[15] Paavilainen L., Edvinsson Å., Asplund A., Hober S., Kampf C., Pontén F., Wester K. (2009). The Impact of Tissue Fixatives on Morphology and Antibody-based Protein Profiling in Tissues and Cells. J. Histochem. Cytochem..

[16] St-Laurent J., Boulay M.-E., Prince P., Bissonnette E., Boulet L.-P. (2006). Comparison of cell fixation methods of induced sputum specimens: An immunocytochemical analysis. J. Immunol. Methods..

[17] Tanaka K.A.K., Suzuki K.G.N., Shirai Y.M., Shibutani S.T., Miyahara M.S.H., Tsuboi H., Yahara M., Yoshimura A., Mayor S., Fujiwara T.K. (2010). Membrane molecules mobile even after chemical fixation. Nat. Methods.

[18] Karnovsky M. (1964). A Formaldehyde-Glutaraldehyde Fixative of High Osmolality for Use in Electron Microscopy. J. Cell Biol..

[19] Kiernan J.A. (2000). Formaldehyde, Formalin, Paraformaldehyde and glutaraldehyde: What they are and what they do. Microsc. Today.

[20] Hopwood D. (1972). Theoretical and practical aspects of glutaraldehyde fixation. Histochem. J..

[21] Huebinger J., Spindler J., Holl K.J., Koos B. (2018). Quantification of protein mobility and associated reshuffling of cytoplasm during chemical fixation. Sci. Rep..

[22] Suthipintawong C., Fcap A., Vinyuvat S. (1996). Immunostaining of Cell Preparations: A Comparative Evaluation of Common Fixatives and Protocols. Diagn. Cytopathol..

[23] Richter K.N., Revelo N.H., Seitz K.J., Helm M.S., Sarkar D., Saleeb R.S., D’Este E., Eberle J., Wagner E., Vogl C. (2018). Glyoxal as an alternative fixative to formaldehyde in immunostaining and super-resolution microscopy. EMBO J..

[24] Alshammari M.A., Alshammari T.K., Laezza F. (2016). Improved Methods for Fluorescence Microscopy Detection of Macromolecules at the Axon Initial Segment. Front. Cell. Neurosci..

[25] Vekemans K., Rosseel L., Wisse E., Braet F. (2004). Immuno-localization of Fas and FasL in rat hepatic endothelial cells: Influence of different fixation protocols. Micron.

[26] Celie J.W.A.M., Beelen R.H.J., van den Born J. (2005). Effect of fixation protocols on in situ detection of L-selectin ligands. J. Immunol. Methods.

[27] Jamur M., Oliver C. (2010). Cell Fixatives for Immunostaining. Methods Mol. Biol..

[28] Melan M. (1999). Overview of Cell Fixatives and Cell Membrane Permeants. Methods Mol. Biol..

[29] Connelly M., Chakraborty U., Brooks H. (1997). Cell fixative and method of analyzing virally infected cells. Biotechnol. Adv..

[30] Li A., Qin Y., Gao M., Jiang W., Liu B., Tian X., Gong G. (2020). Protocol for Imaging of Mitoflashes in Live Cardiomyocytes. STAR Protoc..

[31] Rocha A.G., Franco A., Krezel A.M., Rumsey J.M., Alberti J.M., Knight W.C., Biris N., Zacharioudakis E., Janetka J.W., Baloh R.H. (2018). MFN2 agonists reverse mitochondrial defects in preclinical models of Charcot-Marie-Tooth disease type 2A. Science.

[32] Valente A.J., Maddalena L.A., Robb E.L., Moradi F., Stuart J.A. (2017). A simple ImageJ macro tool for analyzing mitochondrial network morphology in mammalian cell culture. Acta Histochem..

[33] Presley A.D., Fuller K.M., Arriaga E.A. (2003). MitoTracker Green labeling of mitochondrial proteins and their subsequent analysis by capillary electrophoresis with laser-induced fluorescence detection. J. Chromatogr. B.

[34] Caro A.A., Bell M., Ejiofor S., Zurcher G., Petersen D.R., Ronis M.J.J. (2014). N-acetylcysteine inhibits the up-regulation of mitochondrial biogenesis genes in livers from rats fed ethanol chronically. Alcohol Clin. Exp. Res..

[35] Brock R., Hamelers I.H.L., Jovin T.M. (1999). Comparison of fixation protocols for adherent cultured cells applied to a GFP fusion protein of the epidermal growth factor receptor. Cytometry.

[36] Fox C.H., Johnson F.B., Whiting J., Roller P.P. (1985). Formaldehyde fixation. J. Histochem. Cytochem..

[37] Thavarajah R., Mudimbaimannar V., Elizabeth J., Rao U., Ranganathan K. (2012). Chemical and physical basics of routine formaldehyde fixation. J. Oral Maxillofac. Pathol..

[38] Okuda K., Urabe I., Yamada Y., Okada H. (1991). Reaction of glutaraldehyde with amino and thiol compounds. J. Ferment. Bioeng..

[39] Smith J.E., Reese T.S. (1980). Use of Aldehyde Fixatives to Determine the Rate of Synaptic Transmitter Release. J. Exp. Biol..

[40] Sabatini D., Bensch K., Barrnett R. (1963). Cytochemistry and electron microscopy. The preservation of cellular ultrastructure and enzymatic activity by aldehyde fixation. J. Cell Biol..

[41] Stanly T.A., Fritzsche M., Banerji S., García E., Bernardino de la Serna J., Jackson D.G., Eggeling C. (2016). Critical importance of appropriate fixation conditions for faithful imaging of receptor microclusters. Biol. Open.

[42] Ochs M., Mühlfeld C. (2013). Quantitative microscopy of the lung: A problem-based approach. Part 1: Basic principles of lung stereology. Am. J. Physiol. Lung Cell. Mol. Physiol..

[43] Li Y., Almassalha L.M., Chandler J.E., Zhou X., Stypula-Cyrus Y.E., Hujsak K.A., Roth E.W., Bleher R., Subramanian H., Szleifer I. (2017). The effects of chemical fixation on the cellular nanostructure. Exp. Cell Res..

[44] Jalali M., Saldanha F., Jalali M. (2017). Basic Science Methods for Clinical Researchers.

[45] Dimitriadis G.J. (1979). Effect of detergents on antibody-antigen interaction. Anal. Biochem..

[46] Jamur M.C., Oliver C. (2009). Permeabilization of cell membranes immunocytochemical methods and protocols. Methods Mol. Biol..

[47] Koley D., Bard A.J. (2010). Triton X-100 concentration effects on membrane permeability of a single HeLa cell by scanning electrochemical microscopy (SECM). Proc. Natl. Acad. Sci. USA.

[48] Schnell U., Dijk F., Sjollema K.A., Giepmans B.N.G. (2012). Immunolabeling artifacts and the need for live-cell imaging. Nat. Methods.

