# Supplementary material for: The Combination of Paraformaldehyde and Glutaraldehyde Is a Potential Fixative for Mitochondria
Source: Biomolecules. 2021 May 10;11(5):711. doi: 10.3390/biom11050711 (PMC8151741; doi:10.3390/biom11050711)
Supplement: Supplementary file 1 [file biomolecules-11-00711-s001.zip › biomolecules-1195560-supplementary.pdf]

Supplementary Materials

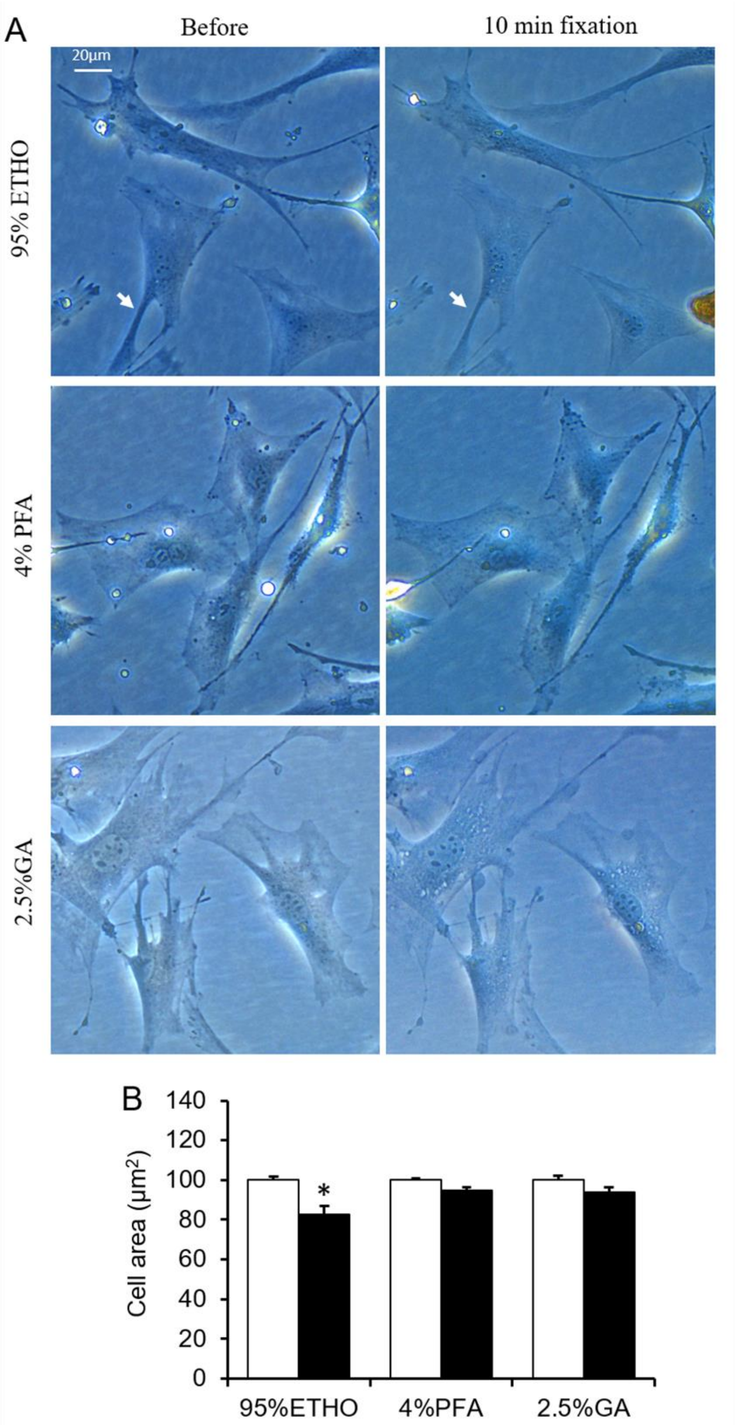

Figure S1. Cell morphology after fixation. (**A**) Representative images of the cell morphology after fixed by PFA, GA, or ETHO. (**B**) Quantitative data of cell area after fixation. Mean ± SEM, n = 12–15. * $p < 0.05$. The data analyzed by *t*-test.

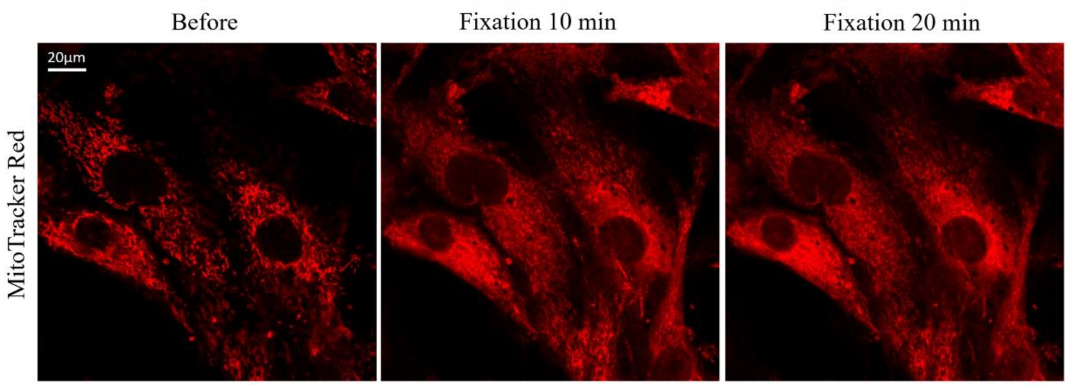

Figure S2. Alternation of MitoTracker Red fluorescence after PFA fixed cells. Representative images of the MitoTracker Red fluorescence in cells fixed at different times by PFA.

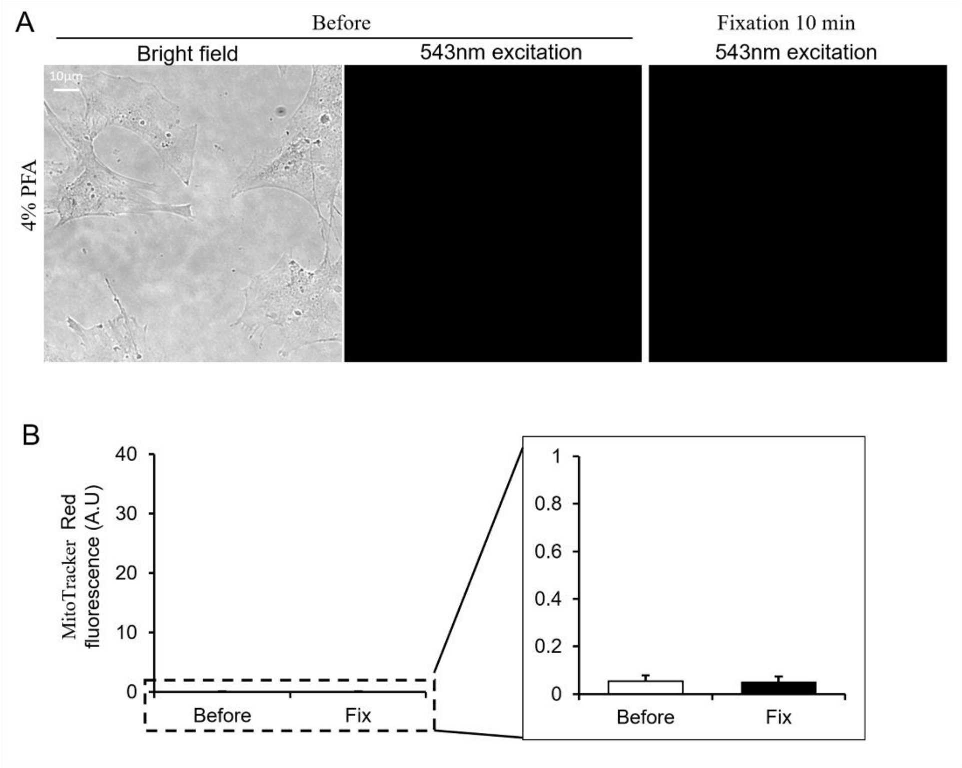

Figure S3. Alternation of autofluorescence in cells after PFA fixed cells. (**A**) Representative images of the bright field and autofluorescence at 543 nm excitation in cells. (**B**) Quantitative data of autofluorescence. Right panel, expanded par figure. Mean ± SEM, n = 9 cells.

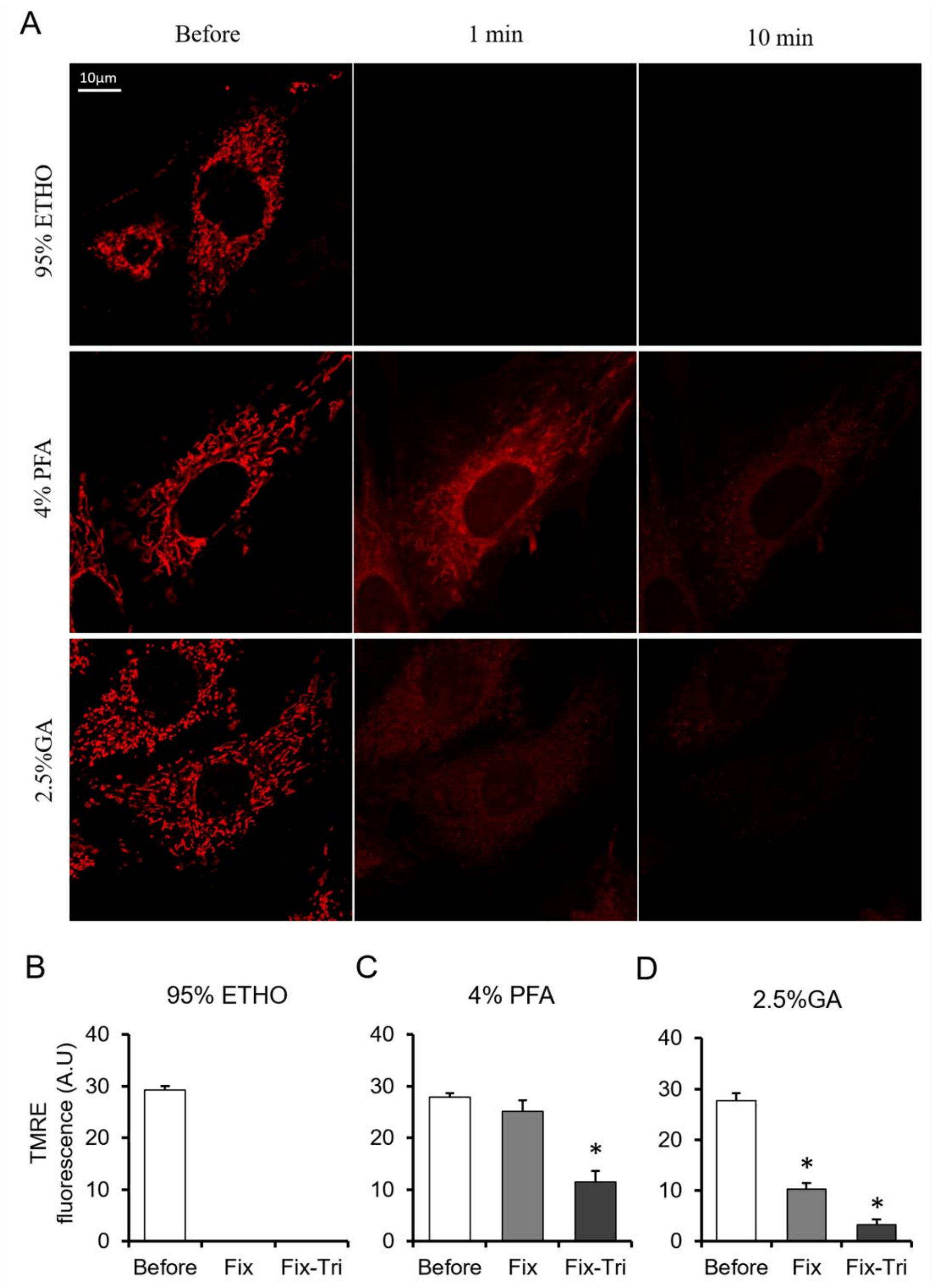

Figure S4. TMRE fluorescence assay on fixed and permeabilized cells. (**A**) Representative images of the TMRE fluorescence after fixed by PFA, GA, or ETHO, and permeabilized by Triton. (**B**–**D**) Quantity results of TMRE fluorescence before and after fixed by ETHO, FA or GA, and permeabilization. Fix, fixation; Fix-Tri, fixation followed with Triton. Mean ± SEM, n = 9. * $p < 0.05$. The data analyzed by one-way ANOVA.

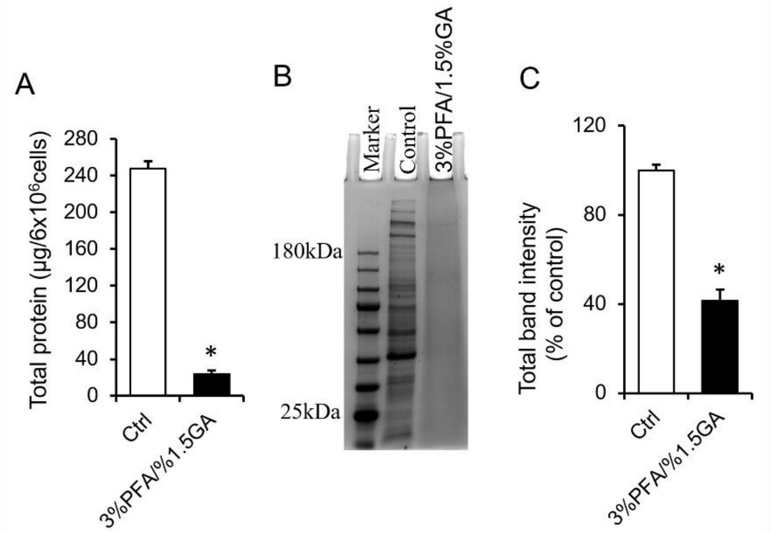

Figure S5. Protein extraction and analysis after PFA-GA fixation. (**A**) The total proteins extracted from fixed cells by PFA-GA. Mean ± SEM, n = 3. * $p < 0.05$. (**B**) Representative images of the SDA-PAGE gel. (**C**) Quantity data of the bands of total protein in PAGE gel. Mean ± SEM, n = 3. * $p < 0.05$. The data analyzed by one-way ANOVA.

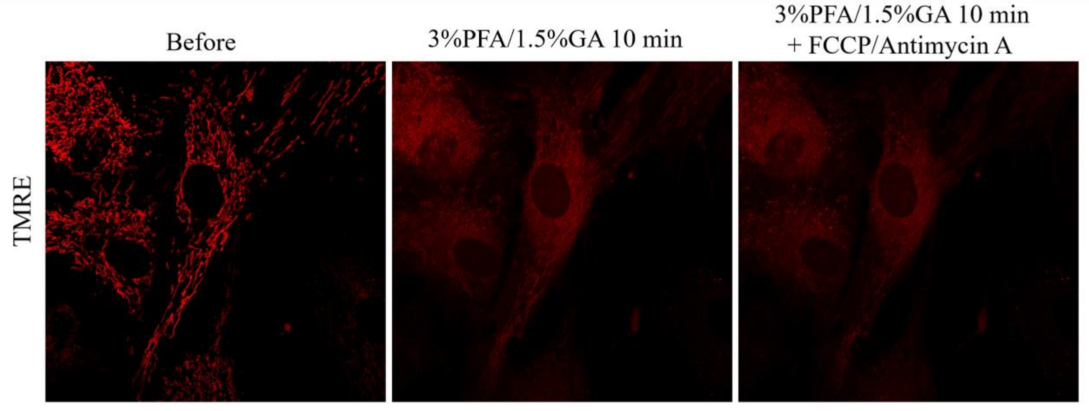

Figure S6. Alternation of TMRE fluorescence in cells. Representative images of the TMRE fluorescence in cells treated by 3% PFA/1.5% GA and FCCP/Antimycin A.

Table S1 The combinations of fixatives.

Chemical / Group	Formaldehyde (%)	Glutaraldehyde (%)	Ethanol (%)
1	4		
2		2.5	
3			95
4	1	1	
5	1	1.5	
6	1	2	
7	1	2.5	
8	2	1	
9	2	1.5	
10	2	2	
11	2	2.5	
12	3	1	
13	3	1.5	
14	3	2	
15	3	2.5	
16	4	1	
17	4	1.5	
18	4	2	
19	4	2.5	

Table S2 The sequence of Q-PCR primers.

Genes	Forward primer	Reverse primer
NRF1	CTTCATGGAGGAGCACGGAG	CTGCCGTGGAGTTGAGGATG
NRF2	TATCCAGGGCAAGCGACTCA	CCCAGCAGGACATGGATTTGA
GAPDH	AGGTCGGTGTGAACGGATTTG	TGTAGACCATGTAGTTGAGGTCA
parkin	GAGGGATTCAGAAGCAGCCA	CACCACTCATCCGGTTTGGA
LC3	CTTGCCTGTCTGCCCATCTT	TCCATACACCGAGTCAGGACA
dynein	CATCATGGTCTTGTCCGATGAG	AGCATCATAGGGTTCATTGCATT
Miro1	TGGGCAGCACTGATAGAATAGA	GCAAAGACCGTAGCACCAAAG
Miro2	CTGGTCGGCGAGGAGTTTC	CCGCTTCTGAGTAATCCACGA
Milton	ACACACCGTGCTTTTCCATGA	CCTTTGTGGAGCCGATGTTC
MFN1	AGCCAAGGAAGTTCTCAACTC	GCTCTGATAGTGTGCTGTTCA
MFN2	TTCTTCGTGCTCGCCAAGG	GCTGCTCAAATTTGGTCTTTACT
P62	CTCCACCAGAAGATCCCAATG	CTGCTTGGCTGAGTGTTACT
PGC1a	GGGCCAAACAGAGAGAGAGG	GTTTCGTTCGACCTGCGTAA
TFAM	GAGCAGCTAACTCCAAGTCAG	GAGCCGAATCATCCTTTGCCT
